# Editorial: Measurements of cardiorespiratory coupling applied to sports and rehabilitation medicine: insights, implications and perspectives

**DOI:** 10.3389/fnetp.2024.1370452

**Published:** 2024-01-24

**Authors:** Raphael Martins de Abreu, Victor Ribeiro Neves, Beatrice Cairo

**Affiliations:** ^1^ Department of Physiotherapy, LUNEX University, International University of Health, Exercise and Sports, Differdange, Luxembourg; ^2^ LUNEX ASBL Luxembourg Health and Sport Sciences Research Institute, Differdange, Luxembourg; ^3^ Department of Physiotherapy, University of Pernambuco (UPE), Petrolina, PE, Brazil; ^4^ Department of Biomedical Sciences for Health, University of Milan, Milan, Italy

**Keywords:** autonomic nervous system, heart rate variability, blood pressure variability, cardiorespiratory coupling, respiratory dynamics, physical performance, sports medicine, cardiovascular oscillations

Comprehensive understanding of the non-invasive study of the parasympathetic and sympathetic nervous systems can be achieved through cardiorespiratory coupling (CRC) analysis. CRC involves the simultaneous examination of heart period (HP) and respiratory oscillations (Dick et al., 2014). This is particularly beneficial given the strong influence of respiration on HP, with shared inputs, common rhythms, and complementary functions exist between these physiological systems. Therefore, the joint study of HP and respiratory oscillations provides valuable insights into the complex relationship between cardiovascular and respiratory functions. Notably, studies have indicated that chronic physical exercise can impact CRC in athletes, which might be associated with individual exercise capacity, such as the maximal oxygen uptake (VO_2_max) (Abreu et al., 2024). An elevated resting CRC has been associated with improved VO_2_max during maximal exercise, highlighting its potential as an indicator of enhanced cardiopulmonary performance. Furthermore, CRC indexes have been associated with the level of qualification of the athletes, as well as post-competition recovery capacity and adaptations to hypoxemic environments (Abreu et al.)

Additionally, in the future, application of CRC analysis could extend to guiding exercise prescription, enabling sports medicine and health professionals to design exercise protocols that may enhance CRC and, subsequently, cardiopulmonary fitness in physiological and pathological conditions. In essence, understanding and utilizing CRC in sports medicine and rehabilitation can significantly contribute to optimizing exercise capacity and understanding the physiological responses during recovery, guiding tailored interventions (Abreu et al.). Therefore, this Research Topic aims to expand our knowledge on the different non-invasive approaches and models for estimating CRC using network-based measures considering the oscillations of cardiovascular and respiratory time series at rest and/or during induced physiological stresses. Furthermore, our focus is on the relationship between CRC indexes and health outcomes in pathological conditions, as well as physical performance in elite and recreational athletes. The width of applications of CRC in sports and clinical settings is evidenced by the contributions in this Research Topic.

CRC can also be studied to evaluate the effects on physiological coupling of enhanced external counterpulsation (EECP), an auxiliary circulation technique used in the rehabilitation of cardiovascular and cerebrovascular diseases: in Liu et al., phase synchronization indexes between respiratory and cardiovascular systems and cardiac and cardiovascular systems were calculated in order to assess the cardiorespiratory and cardiovascular profiles of healthy subjects undergoing EECP or sham treatment.

Respiratory manoeuvres with change in breathing rate can also be employed in sports medicine and medical rehabilitation with prognostic value: in Romanchuk the assessment of cardiorespiratory parameters was carried out in athletes using a spiroarteriocardiograph, in order to evaluate breathing pattern and volume variability, heart rate and blood pressure variability, hemodynamics and cardiorespiratory system synchronization.

Rodriguez et al. proposed a novel method based on the influence of cardiorespiratory activity on blood pressure to classify cardiomyopathy patients. The three-dimensional representation of cardiorespiratory and vascular activities revealed distinct patterns in dilated cardiomyopathy (DCM) patients’ respiratory response to decreasing blood pressure, while ischemic cardiomyopathy (ICM) patients exhibited more stable cardiorespiratory activity. Overall, cardiomyopathy patients displayed limited regulatory capacity for blood pressure changes. Support vector machine models, based on the best classifiers, achieved high accuracy, sensitivity, and specificity in distinguishing between ICM, DCM patients, and control subjects. This innovative approach holds promise for early prognosis and targeted interventions in patients with cardiomyopathies.

The works collected in the present Research Topic contribute to better define and advance the state of the art on CRC assessment in sports and rehabilitation medicine, by applying diverse evaluation methodologies and approaches in a variety of settings and populations.
